# Association between Single Nucleotide Polymorphisms and Endometriosis in a Brazilian Population

**DOI:** 10.1055/s-0040-1708460

**Published:** 2020-03

**Authors:** Paula Coelho Silva Viana, Ana Carolina Delgado Malvaccini Mendes, Lucas Farah Delgado, Gustavo Tostes, Lidiane Gonçalves, Homero Gonçalves Júnior, Nádia Rezende Barbosa Raposo, Geraldo Sérgio Farinazzo Vitral, Pamela Souza Almeida Silva Gerheim

**Affiliations:** 1 Universidade Federal de Juiz de Fora, Campus Governador Valadares, Governador Valadares, MG, Brazil; 2 Universidade Federal de Juiz de Fora, Campus Juiz de Fora, Juiz de Fora, MG, Brazil

**Keywords:** endometriosis, polymorphism, single nucleotide, genetics, endometriose, polimorfismo de nucleotídeo único, genética

## Abstract

**Objective** To investigate the association between genetic polymorphisms in candidate genes or candidate regions and the development of endometriosis in Brazilian women.

**Methods** A total of 30 women between 25 and 64 years old with a diagnosis of endometriosis participated in the present study, as well as 30 matched control women from the same age group, asymptomatic and without family history of the disease. The patients genotypic and allelic frequencies of polymorphisms in the *GREB1* gene (rs13394619) and in the intergenic region at position 7p15.2 (rs12700667) were analyzed and compared.

**Results** There was no significant difference in the frequency of genotypes for the A > G polymorphism (rs13394619) in the *GREB1* gene between the two groups. However, the distribution frequencies of the genotypes for the A > G polymorphism (rs12700667) in an intergenic region on chromosome 7 were different for control patients and for patients with endometriosis, with higher frequency of the AG genotype compared to the GG between patients with the disease (odds ratio [OR] = 3.49; confidence interval [CI] = 1.47–8.26).

**Conclusion** The present study suggests that the polymorphism in the intergenic region of chromosome 7 is associated with the risk of developing endometriosis in a population of Brazilian women from Juiz de Fora.

## Introduction

Endometriosis (ICD 10–N80) is defined by the presence of endometrial glands or stroma outside the uterine cavity. Dysmenorrhea, dyspareunia and chronic pelvic pain are symptoms of the disease, whose intensities are not necessarily proportional to the endometriotic process extension. The gold standard method for definitive diagnosis is laparoscopy with biopsy or visualization of the endometriotic foci.^1^

Early menarche, late pregnancies and large time difference between menarche and first pregnancy are risk factors for the development of endometriosis.^2^ Its prevalence is higher among Caucasian and Asian women compared to Hispanic and black women.^1^ Among reproductive-age women, the global prevalence of the disease is ∼ 10 to 20%, and infertility affects ∼ 30 to 50% of those patients.^2^

The risk of endometriosis is known to be influenced by genetic factors, with an approximate heritability of 51%.^3^ Although the genes involved in the process are not completely known, a portion of the genetic variations already studied are located in genes related to growth factor systems, inflammatory and immune mediators, steroid synthesizing and detoxification enzymes, hormone receptors, apoptosis-linked pathways, among others.^4^ ^5^

In order to identify genetic risk factors related to the development and severity of endometriosis, Genome-Wide Association Studies (GWAS) were performed in different populations and highlighted important associations between single nucleotide polymorphisms (SNPs) and the disease.^6^ ^7^ ^8^ Since then, several studies have been developed seeking to replicate the results found.

Thus, the aim of the present study was to investigate the association of genetic polymorphisms (rs12700667 and rs13394619) in candidate genes or regions from GWAS with the presence of endometriosis in a Brazilian population of women treated at the Juiz de Fora University Hospital.

## Methods

This case-control study was conducted from August 2014 to December 2017 respecting the protocol approved by the Research Ethics Committee of the Universidade Federal de Juiz de Fora (UFJF, in the Portuguese acronym) (CAAE: 30335714.7.3001.5133) and informed consent was obtained from all of the recruited participants.

The case group was composed by 30 women aged between 25 and 64 years old undergoing laparoscopy or laparotomy, who had been histologically diagnosed with endometriosis at any location or stage. These patients were followed by the UFJF University Hospital Videolaparoscopy Outpatient Clinic. Exclusion criteria for this group were diagnosis of adenomyosis, endometrioid carcinoma or ovarian clear cell carcinoma.

The control group consisted in 30 women aged between 25 and 64 years old, who were asymptomatic and undergoing routine preventive examinations in the Gynecology Service of the UFJF University Hospital. Exclusion criteria defined for the control group were personal history of chronic pelvic pain and/or secondary dysmenorrhea and/or abnormal uterine bleeding; personal history of uterine myoma and/or breast cancer and/or benign or malignant ovarian tumors; and family history of endometriosis. The aim of the criteria was to reduce the risk of women in the control group having undiagnosed endometriosis.

After that, individual interviews were conducted and the collected data and information were later transferred to a form created by the researchers. The evaluated parameters were weight, age, height, race, habits (smoking and drinking), comorbidities, medications used, as well as information about endometriosis, when applicable (such as time of diagnosis, treatment, medications, etc.). The form also included hematological data obtained from collection on the day of recruitment.

The DNA necessary for genetic analyzes was extracted from the leukoplatelet layer obtained from the whole blood of the patients using the extraction kit QiaAmp DNA Blood mini kit (Qiagen, Hilden, Germany). Genotyping was performed with ThermoFisher standardized assays for SNPs. The first evaluated SNP is located in an intergenic region in position 7p15.2 (rs12700667) and was analyzed by the presence of G or A allele (Assay ID: C__27263270_10). The second evaluated SNP is located in the 2p25 region in the growth regulation by estrogen in breast cancer 1(*GREB1*) gene (rs13394619) and the presence of G or A allele was also de focus of analyzes (Assay ID: C__11794007_10). The reactions were performed with the aid of StepOne Plus Real Time (ThermoFisher, Waltham, MA, USA) and genotypic analyzes were performed using the software Allelic Discrimination from Applied Biosystems (Foster City, CA, USA).

The clinical and laboratory characteristics of the patients were compared between the endometriosis group and the control group by unpaired t-test (parametric data), Mann Whitney (non-parametric data) and X2 or Fisher test when appropriate (categorical variables). Differences in allele/genotype distribution between the groups were assessed by the X2 Test. The distribution of genotypes for each polymorphism was assessed for deviation from the Hardy-Weinberg equilibrium. The strength of the association between alleles/genotypes and groups was assessed by calculating odds. All statistical analyzes were performed using GraphPad Prism version 6.01 software (GraphPad Software, San Diego, CA, USA), considering *p* < 0.05 as significant.

The formula described by Fleiss et al^9^ was used to calculate the statistical power. For our sample size, the statistics showed a power of 80% to detect differences, with 95% confidence, between the 2 groups with effect sizes (odds ratio [OR]) ≥ 4.5, considering a reference allele frequency between 30 and 40%. When the OD was 3.49, the statistical power was 65%. The data were analyzed using the software R version 3.6.1 (R Core Team, 2019).

## Results

The characterization of patients at the UFJF University Hospital included in the present study is shown in Table 1. Patients with endometriosis had lower levels of hemoglobin and hematocrit than patients in the control group (*p *< 0.05) (Table 1).

**Table 1:** Characteristics of the studied groups

Character	Controls (n = 30)	Endometriosis (n = 30)	*p-value*
Age (years old)	45.2 ± 12.5	36.7 ± 8.1	**0.002** ^a^
Skin colour (white, %)*	40	27	0.270^b^
Red blood cell, 10^6^/L	4.71 ± 0.50	4.60 ± 0.52	0.404^a^
Haemoglobin, g/dL	13.54 ± 1.09	12.90 ± 1.11	**0.024** ^b^
Hematocrit, %	41.48 ± 3.62	39.12 ± 3.26	**0.003** ^c^
Leucocyte count, x10^3^/L	8.13 ± 2.03	7.71 ± 2.32	0.346^c^
Platelet count, 10^5^x mm^3^	2.47 ± 0.52	2.64 ± 0.82	0.563^c^

Values are expressed as mean values ± standard deviations. The values in bold indicate statistically significance of *p* < 0.05. * Self-reported skin colour (as a surrogate for race based on Brazilian census categories). The analyses were developed using: ^a^the unpaired t-test; ^b^the chi-square test; ^c^Mann-Whitney.

Table 2 shows the distribution of genotypes and alleles for *GREB1* and intergenic region genetic polymorphisms. No deviations from the Hardy-Weinberg equilibrium was found (*p* > 0.05; *X* ^2^ = 0.74). There was no significant difference in the frequency of genotypes for the A > G polymorphism (rs13394619) in the *GREB1* gene between the two groups (Table 2). However, the distribution frequencies of the genotypes for the A > G polymorphism (rs12700667) in an intergenic region on chromosome 7 were different between controls and patients with endometriosis (*p* = 0.013; Table 2). The GA genotype frequency was 33% in the controls and 50% in the cases. Compared with the GG genotype, the GA genotype was associated with a significantly increased risk of developing endometriosis (OR 3.49; 95%CI: 1.47–8.26) (Table 2).

**Table 2:** Frequencies of two SNPs and association with endometriosis

Gene/ SNP	Genotypes /Alleles	All % (*n*)	Controls % (*n*)	Cases % (*n*)	*p-value^a^*	OR (95%CI)
*GREB1*	AA	42 (25)	43 (13)	40 (12)	0.428	1.00
rs13394619	AG	42 (25)	43 (13)	40 (12)		1.00 (0.54–1.84)^b^
	GG	17 (10)	13 (4)	20 (6)		1.65 (0.73–3.76)^b^
	A	63 (75)	65 (39)	60 (36)	0.559	1.00
	G	38 (45)	35 (21)	40 (24)		1.24 (0.70–2.20)^c^
Intergenic	GG	17 (10)	23 (7)	10 (3)	0.013	1.00
region	GA	42 (25)	33 (10)	50 (15)		3.49 (1.47–8.26)^d^
rs12700667	AA	42 (25)	43 (13)	40 (12)		2.14 (0.91–5.05)^d^
	G	38 (45)	40 (24)	35 (21)	0.465	1.00
	A	63 (75)	60 (36)	65 (39)		1.24 (0.59–2.60)^e^

Abbreviations: CI, confidence interval; OR odds ratio.

a The analyses were developed using the chi-square test. Bold values indicate that the values have statistical significance. ^b^The odds ratio of the AG and GG genotypes against the AA genotype for rs13394619. ^c^The odds ratio of the A allele against the G allele for rs13394619. ^d^The odds ratio of the AG and AA genotypes against the GG genotype for rs12700667. ^e^The odds ratio of the G allele against the A allele for rs12700667.

## Discussion

The original GWAS, as well as meta-analyses subsequently performed, evidenced important associations between SNPs and the development and severity of endometriosis in several populations.^7^ ^10^ The highlighted polymorphisms include rs13394619 in *GREB1* gene and rs12700667 in intergenic region in 7p15.2 position, which had their frequency and association to the occurrence of endometriosis evaluated in women from Juiz de Fora assisted at the University Hospital.

In our study, for both SNPs evaluated, the allelic frequencies found in the patients (Table 2) were close to the values described for the global population in accordance with the Genome Aggregation Database (Appendix 1), which assembles sequences of more than 140 thousand people from several parts of the world. The frequencies of these alleles present great variability according to ancestry (Appendix 1). For the polymorphism in the *GREB1* gene, the frequency of the G allele is higher in Caucasian Europeans than among Africans. On the other hand, in the intergenic region, the G allele is more common in Africans and Asians than in Europeans and Americans. Since Brazil is marked by the presence of a highly admixed population, being skin color and other phenotypic characteristics weak predictors of genomic ancestry, the studies that assess the distribution of genotypes for polymorphisms in *GREB1* genes and in the intergenic region provide important genetic information concerning this specific population.^11^

**Appendix 1:** Minor alleles frequencies of the studied polymorphisms from populations of different ethnic origins, obtained from HapMap, gnomAD – Genomes and 1000Genomes database (dbSNP)

Polymorphism	Population	Study		
		HapMap	gnomAD-Genomes	1000 genomes
GREB1* (rs13394619)	Global	−	0.41	0.38
	European	0.53	0.52	0.47
	African	0.06	0.14	0.08
	Asian	0.48	0.48^a^	0.51^a^
	American	−	0.44	0.41
Intergenic region* (rs12700667)	Global	−	0.39	0.54
	European	0.24^b^	0.27	0.28
	African	0.64^c^	0.56	0.62
	Asian	0.81^d^	0.84	0.82^a^
	American	−	0.40	0.36

*G allele was considered less frequent; ^a^East Asian ancestry; ^b^Caucasian European ancestry; ^c^YRI: Yoruba Sub-Saharan African ancestry; ^d^HCB: Han Chinese ancestry.

Additionally, our data suggests association between rs12700667 and endometriosis diagnosed by laparoscopy in a Brazilian population, from the city of Juiz de Fora, state of Minas Gerais, being the AG genotype more frequent among patients with endometriosis than among control patients (Table 2). Such finding corroborates those recently found by Li and colleagues who identified the increased risk of endometriosis in patients with the AG genotype in relation to those with the GG genotype in a population in the north of China (OR = 1.57; 95%CI =1.23–2.00; *p* = 0.002).^12^

The first study to highlight the relation between rs12700667 and endometriosis was the GWAS performed in 2011 by Painter et al,^7^ which included 3,194 patients with surgical diagnosis of the disease and other 7,060 control patients from Australia and the United Kingdom, besides a total sample of 4,663 American patients for replication. For any degree of endometriosis, classified according to the American Fertility Society, there was a relation between the prevalence of endometriosis and the frequency of risk allele A for rs12700667. In 2012, Nyholt et al^10^ performed a meta-analysis including, besides the aforementioned GWAS, the GWAS performed by Uno et al in 2010^6^ with a Japanese sample of 1,423 cases and 1,318 controls, along with other 484 cases and 3,974 controls for replication.^6^ ^10^ Although the Japanese study had not pointed allele A for rs12700667 as relevant for endometriosis initially, the meta-analysis showed that the outcome found among Europeans was valid among the Japanese as well, producing a total OR of 1.22 (95%CI: 1.14–1.30).^10^

In 2013, a GWAS conducted in a European population did not point to significant evidence for rs12700667, as well as other replication studies in specific populations.^8^ ^13^ However, other replication studies found correlation between the referred polymorphism and endometriosis in Chinese and Polish populations, as well as the meta-analysis that included GWAS in a Belgian population.^12^ ^14^ ^15^ Thus, our results corroborate the majority of previous studies, suggesting that the rs12700667 is relevant to the development of endometriosis not only in European and Asian populations, but also in a highly admixed population such as the Brazilian.

On the other hand, no associations between A > G (rs13394619) polymorphism in the *GREB1* gene and endometriosis in the present study were found, being the genotypic and allelic frequencies similar between the case and control groups (Table 2). Similarly, a recent study with a Polish sample of 315 infertile women with endometriosis and 406 healthy fertile women did not find any differences in the distribution frequencies of this polymorphism between the groups.^14^

In 2010, the GWA study by Adachi et al validated an important association between endometriosis and A > G (rs13394619) polymorphism in the *GREB1* gene of the 2p25.1 chromosome (OR = 1.35; 95%CI= 1.06–1.18; *p *= 3.8 × 10^−5^).^16^ In 2012, this association was also found (OR = 1.12; 95%CI =1.06–1.18; *p *= 2.1 × 10^−5^) in the meta-analysis that comprehended Japanese and European populations.^10^ When searching to replicate the results, a study in a Belgian population found association between rs13394619 and endometriosis at any stage, classified in accordance with the American Fertility Society. Nevertheless, the association was not considerable when only stages III and IV and, thus, more aggressive of endometriosis, were considered, which suggests relation of the polymorphism with the initial stages of the disease.^15^ Another study assessing the relation between *GREB1* with endometriosis verified the relevance of rs13394619, although various other polymorphisms in the same gene had superior significance, suggesting greater relevance in the development and severity of endometriosis.^17^

Although rs13394619 is an endometriosis susceptible SNP identified in large and significant studies, the association was not replicated in our study, which might be related to our small population. This inconsistency happens in relation to many other SNPs in the literature, also evidencing the importance of ethnic differences, which can result in different genetic influences. The small sample aforementioned is an important limitation of our study, which can lead to false associations. It is also important to point out the fact that patients with endometriosis might have been included in the control group, even though they presented no clinical evidence of such, since laparoscopy exams to rule out the disease were not performed in every patient.

## Conclusion

The present study is a pioneer when it comes to analyzing the frequency of two SNPs in a Brazilian population with endometriosis, being the first survey to present this data referring to a population in the state of Minas Gerais. As the frequency of such polymorphisms varies a lot according to ancestry, such findings are important to provide information about an admixed population. Additionally, the frequency of the AG genotype for rs12700667 in the intergenic region in chromosome 7 was higher among patients with endometriosis than among control patients, suggesting that such polymorphism could be related to the development of the disease. Nonetheless, additional studies broadening this sample will be important to confirm such findings.
